# The effect of intrauterine human chorionic gonadotropin injection before embryo transfer on the implantation and pregnancy rate in infertile patients: A randomized clinical trial

**Published:** 2016-10

**Authors:** Razieh Dehghani Firouzabadi, Sima Janati, Mohammad Hossein Razi

**Affiliations:** 1 *Research and Clinical Center for Infertility, Shahid Sadoughi University of Medical Sciences, Yazd, Iran.*; 2 *Department of Obstetrics and Gynecology, School of Medicine, * *Research and Clinical Center for Infertility, Dezful University of Medical Sciences, Dezful, Iran.*

**Keywords:** *Human chorionic gonadotropin*, *ICSI*, *Embryo transfer*, *Pregnancy outcome*

## Abstract

**Background:**

Implantation is one of the essential steps for the success of assisted reproductive techniques (ART). Their success depends on three main factors: embryo quality, endometrial receptivity (ER), and synchrony between embryo and endometrium. There are various factors that regulate the complex process of implantation. In this regard, one may refer to human chorionic gonadotropin (hCG) as the most important factor.

**Objective:**

This study aims to investigate the effect of intrauterine hCG injection before embryo transfer (ET) on pregnancy outcome in infertile couples.

**Materials and Methods:**

A total of 159 patients undergone In vitro Fertilization/ Intracytoplasmic Sperm Injection (IVF/ICSI) with an antagonist protocol were evaluated. Patients were divided into three groups (n=53). Group 1 received 500 IU of hCG, group 2 received 1000 IU of hCG intrauterine injection before ET, and the control group underwent ET without hCG preceding intrauterine injection.

**Results:**

There was no significant difference among the groups. The implantation rates were 18.86%, 13.52%, and 14.37%, chemical pregnancy rates were 34%, 32.1%, and 35.3%, and clinical pregnancy rates were 34%, 32.1%, and 31.4% respectively.

**Conclusion:**

The pregnancy outcome in IVF/ICSI /ET cycles cannot be improved through hCG intrauterine injections before ET.

## Introduction

One in six to seven couples is affected by infertility in world. Assisted reproductive techniques (ART) are currently accountable for up to 7% of childbirths in developed countries (1). In recent years, despite the major advances in different aspects of clinical and laboratory ART, pregnancy rate still remains low (2-4). Implantation is one of the crucial stages for ART achievement (5, 6). There are three major aspects to determine how successful ART can be including; embryo quality, endometrial receptivity (ER) and embryo-endometrium synchronization (3, 6, 7). 

There are several factors that coordinate the complex process of implantation. The most important factor in this regard is human chorionic gonadotropin (hCG) (3, 8, 9). It is the last consequence of complex molecular interactions between prepared uterus and a mature blastocyst. Unsuccessful implantation is a major restrictive cause in assisted reproduction (10). It is anticipated that approximately 50-75% of pregnancy losses are due to implantation failure (4, 10). There seem to be three components that are essential for establishing a receptive endometrium and initiating an embryo-maternal crosstalk. "These components include ovarian steroid hormones, local autocrine and paracrine signalling in the endometrium, and embryo-derived signals "(5).

The most considerable human embryonic signal that has been known since long and one of the earliest molecules that is secreted by the embryo before its implantation is hCG (4, 8, 9, 11). The embryo’s mRNA is, indeed, determined when it is at the 6-8 cell stage, and the blastocyst produces protein before its implantation (8, 11, 12). HCG production by the syncitiotrophoblast increases after implantation (8). "It is implicated in several actions that promote tolerance and angiogenesis at the maternal-fetal interface has important physiological implications for successful pregnancy" (4, 8).

In order to investigate the direct effects of hCG on the human endometrium for the first time, an intrauterine micro dialysis instrument was created by Licht *et al*. The instrument was also used for measuring paracrine mediators in the uterine cavity. The researchers showed that intrauterine administration of 500 IU of hCG/mL can cause a significant inhibition of intrauterine insulin-like growth factor-binding protein 1 (IGFBP-1) and macrophage colony- stimulating factor (M-CSF). In addition, leukemia inhibitory factor (LIF), which is a cytokine needed for implantation, vascular endothelial growth factor (VEGF), which is a proangiogenic growth factor, and Metalloproteinase-9 (MMP-9), which is a regulator of tissue remodeling, were incited as significant factors (11-13). In another study, Ragga Mansor investigated how valuable intrauterine injection of hCG is before embryo transfer (ET). They showed that intrauterine injection of 500 IU of hCG before ET enhanced IVF/ICSI outcomes significantly (3). 

On the basis of the hypothesis that injection of hCG inside the uterine cavity before ET enhances implantation, this clinical trial aims to investigate the effect of hCG injection before embryo transfer on pregnancy outcome in infertile couples.

## Materials and methods

**Population**

In this prospective, randomized, unblinded, clinical trial study, a total number of 159 patients who were referred to Yazd Research and Clinical Center of Infertility from April 2012 till March 2013 and underwent IVF/ICSI with an antagonist protocol were enrolled. The patients were given sufficient information before they provided written informed consents. The study was approved by the Yazd Research and Clinical Center for Infertility Ethics Committee.

**Randomization**

The defined inclusion criteria were female age in the range of 20-40 years with a male factor or unexplained infertility and basal FSH less than 12. The exclusion criteria were defined as azoospermia, presence of uterine myoma, endometriosis, hydrosalpinges, previous IVF/ICSI trials (successful or unsuccessful), history of endocrine diseases such as diabetes and thyroid dysfunction, previous history of hysterocopic operation due to submoucosal myoma, and intrauterine synechia. Liable women were randomly assigned to two test groups in the ratio of 1:1 and a control group according to computer-generated random numbers (n=53). Group 1 received 500 IU of hCG, group 2 received 1000 IU of hCG intrauterine injection before ET and, in the control group, ET was done without hCG intrauterine injection. 

**Treatment protocols**

One hundred and fifty nine women with a male factor or unexplained infertility underwent IVF/ICSI and were treated with an antagonist protocol. All the patients received low-dose oral contraceptive pills (OCP LD) (30 mg Ethinyl Estradiol and 0.3 mg Norgestrel, Aburaihan Co., Tehran, Iran) from the second day of their menstrual cycle then ceased until menstruation happened. As menses started, gonadotropin stimulation was recommended to be done by means of Gonal-F (Gonal-F, Serono, Aubnne, Switzerland) from the second day of the menstrual period. According to the patients’ age and body weight, the initial dose of gonadotropin was determined to be 150-300 IU/day.

Monitoring was started on day 7 of the stimulation, and the dose of gonadotropin was adjusted according to estradiol (E_2_) serum concentration and ovarian response that were examined by ultrasound. When the leading follicles reached 14 mm in diameter, Cetrorelix (Merck-Serono, Germany) 0.25 mg was subcutaneously added and repeated every day till the day of hCG administration. An intramuscular (IM) injection of 10,000 IU of hCG (Pregnyl®, Organon, Oss, Netherlands) was done as the minimum three follicles reached a diameter of ≥18 mm. Ovum pickup was performed by the ultrasound guide 36 hours after the hCG injection.

**Semen analysis and processing before ICSI**

After 3 to 4 days of abstinence, on the day of ovum pick-up, semen samples collection was done by masturbation. Sperm count of each sample was performed in a Makler chamber, and sperm motility was estimated using light microscopy. This estimation conformed with the criteria of the World Health Organization. Also, sperm morphology was studied by wet preparation as the common practice is in semen assessments. It was assessed on the basis of Kruger’s strict criteria within limits of a light microscope magnification of 100 (14). Sperms without midpiece or tail defects, normal shape, head, size and acrosome were considered normal. Using density gradient sperm-separation techniques, the semen samples were rinsed after liquefaction. Then, the final sperm pellet was resuspended in a 0.5 ml supplemented medium. 

**IVF/ICSI procedure**

After retrieval of oocytes, they were washed in a G-MOPS medium (Vitrolife, Sweden), incubated in a culture medium (GIVF-plus; Vitrolife, Sweden), and then covered with a mineral oil (Ovoil; Vitrolife, Sweden) for two hours at 37^o^C and in 6% CO_2_ and 5% O_2_. Cumulus cells were taken away mechanically through a 30-second exposure to the Hyase medium containing 80 IU/mL of hyaluronidase (Vitrolife, Sweden). Then, coronal cells were eliminated gently, and the denuded oocytes were evaluated for any nuclear status. As the mature oocytes were counted, there proved to be the expulsion of the first polar body. The oocytes that represented the release of the first polar body were considered mature and, thus, chosen for ICSI. 

The oocytes which had one, three or more pronuclei or were immature, deformed, and post-mature were eliminated from the study. An Eppendorf micromanipulator assembled on a Nikon inverted microscope was used to perform ICSI. The oocytes were examined 16-18 hours after microinjection to determine whether there were any pronuclei. For this purpose, a Nikon inverted microscope was used. The fertilize oocytes were cultured in-vitro for 2 to 3 days to evaluate their growth and cell division. In order to reduce OHSS risks, all the fresh embryos in good quality were cryopreserved when the patients showed symptoms that were suggestive of OHSS or when the serum estradiol concentration exceeded 4000 pmol/L on the day of hCG administration.

**Evaluation of embryo quality and embryo transfer**

Fertilization and embryo quality were evaluated by a professional embryologist. The Hill’s criteria were used to score the cleavage-stage embryos.(15). High-quality embryos, which lacked fragmentation but had equal-sized homogenous blastomeres (4-cell embryo on day 2 or 8- cell embryo on day 3) and homogeneous cytoplasms were characterized as grade A. Grade B was assigned to embryos with ≤10% fragmentation, and homogenous blastomeres of the same size. Grade C marked embryos with ≤50% fragmentation blastomeres of unequal size, and large granules. Finally, embryos that had >50% fragmentation, blastomeres of unequal size, and large black granules were considered to be of grade D. Grade-A and B embryos were deemed as high-quality ones, and their percentage was computed. Intrauterine injection hCG was prepared by adding 1 ml of tissue culture media (G.2 plus, Vitrolife, Sweden) to a vial of hCG containing 5000 IU hCG (Pregnyl®, Organon, Oss, Netherlands). Then, hCG was diluted as required (500 and 1000 IU) for intrauterine injection.

Embryo transfer was conducted by ultrasound guidance 48-72 hrs after oocyte retrieval. The number of the transferred embryos depended on their quality and the patients’ age. The embryos in excess were frozen in liquid nitrogen using the vitrification method. In groups 1 and 2, after a speculum was inserted and the cervix was viewed in the lithotomy position, 0.04 µl of a tissue culture medium containing 500 or 1000 IU of hCG was passed into the internal cervical OS through a Labotect catheter (Labotect, Gotting, Germany). Approximately, 7 minutes after intrauterine hCG injection, 1-3 embryos were transferred for each couple, using a Labotect catheter (Labotect, Gotting, Germany). In the control group, ET was done without intrauterine hCG injection, just the same way as in groups1 and 2. For all the patients, the residual embryos were frozen in liquid nitrogen by the vitrification method.

Each patient received 100 mg of progesterone (Aburaihan Co., Tehran, Iran) per day in the IM manner and 4 mg of estradiol valerate (Aburaihan Co., Tehran, Iran) per day as a luteal support, starting on the day of oocyte retrieval. Serum β-hCG level was checked 14 days after ET. If it was positive, vaginal or abdominal sonography was performed 2-3 wks later to view the number of gestational sacs and presence of fetal heart action. After pregnancy confirmation, the luteal support was continued until the 10^th^ wk of pregnancy.

**Outcome measures**

An evaluation was made of the primary outcome measures, including the rate of implantation as well as the rates of chemical and clinical pregnancies. The secondary outcome regarded the abortion rate. The percentage of the implantation rate was calculated as the following fraction: gestational sac(s)/ the number of embryos transferred ×100. 

Chemical pregnancy was considered as serum βhCG ≥25 IU/L measured two weeks after ET. However, clinical pregnancy was characterized by the presence of a gestational sac with heart beats identified through vaginal or abdominal ultrasound 4-5 wks after the embryo transfer. The rate of clinical abortion was defined as pregnancy losses that were clinically documented earlier than 20 wks of gestation.

**Statistical analysis**

The SPSS 19 package program (SPSS Inc, IBM) was used to perform all the statistical analyses. One-way analysis of variance test (ANOVA) was used to compare the means of three groups (500 hCG, 1000 hCG, and control).  ^2^ or Fisher exact test was used for qualitative variables as appropriate. A two-sided p<0.05 was considered statistically significant. The paper offers the data as the mean±SD and percentages when appropriate.

## Results

The results were reported in accordance with the CONSORT statement. Of 317 IVF/ICSI candidates, 158 were excluded. A total of 159 patients were enrolled in our study and randomly divided into 3 groups. No follow-up patient lost. However, 2 patients in the control group, embryo transfer were cancelled due to the ovarian hyperstimulation syndrome. No cycle was cancelled due to the failure of embryo fertilization, or of oocyte retrieval, or of access to proper semen samples. Basically, there arose no such problems.

**Figure 1:**
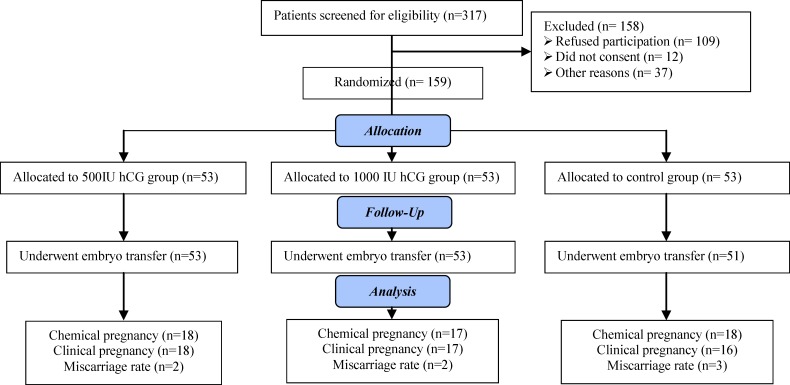
Flowchart of patient selection and pregnancy outcome

The CONSORT statement flow diagram is presented in Figure 1**.** Table I presents the basic and demographic characteristics of the patients in terms of age, basal FSH level, cause, type and duration of infertility. There were no significant differences between the groups (p>0.05). 

The most common cause and type of infertility were male factor and primary infertility respectively in all the groups. Table II presents the cycle characteristics of the groups comparatively. As it can be seen, there was no statistically significant difference between the groups in terms of the number of follicles more than 16 mm, retrieved oocytes and mature oocytes. Moreover, there was just a non-significant difference of the endometrial thickness and the estradiol level found on the day of hCG administration between the study groups on one hand and the control group on the other (p=0.2, p=0.1 respectively).

Table III shows no significant difference in the number of embryos obtained, fertilized oocytes, and frozen embryos among the three groups (p=0.36, p=0.99, and p=0.76 respectively). Also, they were not different in terms of mean number of transferred embryos.

In fact, the data suggest, the mean number and the grade of the transferred embryos, good-quality embryos as well the number of frozen embryos were similar in all the groups. Also, in each group, the mean number of the transferred embryos was two, and the grade of the transferred embryos was mostly B and C, while grade D embryos were not transferred. 

The total cancelation rate was not significantly different between groups (0% in study groups vs. 3.8% in the control) (p=0.5). Table IV presents the pregnancy outcomes in the groups. The statistical analysis revealed no evidence of difference between the groups with regard to the rate of implantation (18.86%, 13.52%, and 14.37% respectively, p=0.51). There was no significant difference between the study groups and the control group with regard to the chemical pregnancy rate (34%, 32.1%, and 35.3% respectively, p=0.94) and the clinical pregnancy rate (34%, 32.1%, and 31.4% respectively, p=0.99). However, no difference was observed between the groups regarding the abortion rate (p=0.83).

When using hCG intrauterine injections, no complications were observed. The mean time between hCG intrauterine injection and ET was 5-7 min, and the timing was similar among all the study groups. 

**Table I:** Basic and demographic characteristics of patients

**Characteristics**		**Group 1**	**Group 2**	**Control group**	**p-value**
Female age (yrs)*		29.39 ± 5.28	29.88 ± 4.9	28.62 ± 4.6	0.87
Basal FSH (IU/L)*		6.64 ± 1.98	6.82 ± 1.89	6.43 ± 2.32	0.61
Duration of infertility (yrs)*		6.5 ± 4.56	7.89 ± 4.8	5.93 ± 4.09	0.07
Infertility etiology**					
	Male factor	48 (90.6%)	51 (96.2%)	50 (94.3%)	0.47
	Unexplained	5 (9.4%)	2 (3.8%)	3 (5.7%)	
Infertility kind**					
	Primary	48 (90.6%)	52 (98.1%)	47 (88.7%)	0.15
	Secondary	5 (9.4%)	1 (1.9%)	6 (11.3%)	

p<0.05 was significant, Anova test, Fisher’s exact test,  ^2 ^test. (n=53)

* Data presented as Mean± SD.

** Data presented as n (%)

**Table II:** Cycle characteristics in studies and control groups

**Characteristics**	**Group 1**	**Group ** **2**	**Control group**	**p-value**
No. of follicles≥16 mm	11.01 ± 3.64	9.94 ± 4.19	10.88 ± 3.88	0.3
No. of oocytes retrieved	10.7 ± 4.67	9.24 ± 4.48	10.2 ± 4.42	0.49
Total MΙΙ oocyte	8.66 ± 3.75	8.22 ± 3.94	8.41 ± 3.83	0.84
Endometrial thickness on day of hCG injection (mm)	8.96 ± 1.72	8.53 ± 1.45	9.08 ± 1.78	0.2
Peak E2 on day of hCG injection (pg/ml)	1444 ± 551.88	1266 ± 855.88	1559 ± 731.08	0.1

Data presented as Mean± SD.

p<0.05 was significant, Anova test. (n=53)

**Table III:** Embryo data of the studies and control groups

**Characteristics**		**Group 1**	**Group ** **2**	**Control group**	**p-value**
No. of embryos obtained*		4.2 ± 2.67	4.5 ± 2.42	4.94 ± 2.88	0.36
No. of fertilized oocytes*		5.2 ± 2.96	5.18 ± 2.57	5.26 ± 2.93	0.99
No. of frozen embryos *		4.57 ± 2	4 ± 1.47	4.48 ± 2.78	0.76
No. of embryos transferred*		2.39 ± 0.74	2.33 ± 0.72	2.34 ± 0.67	0.83
Grade of transferred embryos**				8.96 ± 1.72	
	A	16 (30.2%)	17 (32.1%)	18 (35.3%)	0.81
	B	34 (64.2%)	30 (56.6%)	29 (56.9%)	
	C	3 (5.7%)	6 (11.3%)	4 (7.8%)	

p<0.05 was significant, Anova test, Fisher’s exact test,  ^2 ^test. (n=53)

* Data presented as Mean± SD.

** Data presented as n (%).

**Table IV:** Outcome measures of cycles in studies and control groups

**Characteristics**	**Group 1**	**Group ** **2**	**Control group**	**p-value**
Implantation rate	18.86%	13.52%	14.37%	0.51
Chemical pregnancy/cycle %	18 (34%)	17 (32.1%)	18(35.3%)	0.94
Clinical pregnancy/cycle %	18 (34%)	17 (32.1%)	16 (31.4%)	0.99
Total abortion rate	2 (3.8%)	2 (3.8%)	3 (5.9%)	0.83

Data presented as n (%)

p<0.05 was significant, Anova test, Fisher’s exact test,  ^2 ^test. (n=53)

## Discussion

HCG is a heterodimeric placental hormone that is produced by the blastocyst before embryo implantation and even more after implantation (3, 8, 9). This hormone has a paracrine role that would be of importance during the early stages of implantation. It also plays a classical endocrine role as to rescue the maternal corpus luteum in early pregnancy (5). Recently, several investigators have shown the paracrine role of hCG in the preparation for implantation and regulation of uterine environment (17). hCG is considered as the original signal given by the embryo once immunologic tolerance and angiogenesis are affected by it at the maternal-fetal interface. This issue is, thus, of physiologically significant implications for successful pregnancy (3, 8, 18). Any direct effect of hCG on the uterine cavity would mean the presence of functioning receptors for the hormones in the endometrium (12). 

In this study, we showed that intrauterine injection of 500 IU and 1000 IU of hCG before ET has no impact on the improvement of pregnancy outcomes. Till now, three studies have been done in this regard. The first experiment was done by Licht *et al* in which the direct effect of hCG on the human endometrium were examined (13). They showed significant effects of hCG on some interfering factors in implantation. The second experiment was done by Banerjee and Fazleabas on baboons as the nonhuman primate model for evaluating embryo implantation (19). 

The researchers infused hCG into the uterine cavity in the window of receptivity. The hCG infusion provoked numerous biochemical, morphological, and molecular alterations in the estrogen- and progesterone-primed endometrium. The third study that was done by Mansour Ragga concluded that intrauterine injection of 500 IU of hCG prior to ET significantly improves success rates in IVF/ICSI (3). It is likely that hCG intrauterine injection before ET causes numerous changes in the endometrium as described in the experiments done by Licht, Mansour Ragga, Banerjee and Fazleabas (3, 19). In contrast to previous experiments, the present study came to different findings in that intrauterine hCG injection has no noticeable effect on implantation and pregnancy outcomes. The first explanation may be the smallness of the sample size used in this study. Secondly, since few studies have been ever done on this topic and many factors contribute to the success or failure of implantation, the expected paracrine effects of intrauterine injection of hCG were not achieved. 

## Conclusion

As a conclusion, it may be claimed that intrauterine injection of hCG before ET is of no capability to improve the implantation and pregnancy rates in IVF/ICSI/ET cycles. However, the findings of the study are in contradiction with some other corresponding pieces of research in the literature. Therefore, more studies need to be conducted in this regard.

## Note

***Registration ID in IRCT: IRCT2012112610328N3***
