# Corrigendum to “The effect of intrauterine human chorionic gonadotropin injection before embryo transfer on the implantation and pregnancy rate in infertile patients: A randomized clinical trial” [*Int J Reprod BioMed* 2016; 14: 657-664]

**DOI:** 10.18502/ijrm.v21i1.12688

**Published:** 2023-02-08

**Authors:** Razieh Dehghani Firouzabadi, Sima Janati, Mohammad Hossein Razi

The authors have been informed of an error that occurred on page 657 in which the IRCT code has been entered incorrectly, which should be corrected as: “IRCT2012091310328N3". On behalf of the author, the publisher wishes to apologize for this error. The online version of article has been updated on 31 January 2023 and can be found at https://doi.org/10.18502/ijrm.v14i10.695.

